# Review Article: Unraveling synergistic effects in plasma-surface processes by means of beam experiments

**DOI:** 10.1116/1.4983275

**Published:** 2017-05-10

**Authors:** Achim von Keudell, Carles Corbella

**Affiliations:** Experimentalphysik II, Ruhr-Universität Bochum, Universitystr. 150, D-44801 Bochum, Germany

## Abstract

The interaction of plasmas with surfaces is dominated by synergistic effects between incident ions and radicals. Film growth is accelerated by the ions, providing adsorption sites for incoming radicals. Chemical etching is accelerated by incident ions when chemical etching products are removed from the surface by ion sputtering. The latter is the essence of anisotropic etching in microelectronics, as elucidated by the seminal paper of Coburn and Winters [J. Appl. Phys. **50**, 3189 (1979)]. However, ion-radical-synergisms play also an important role in a multitude of other systems, which are described in this article: (1) hydrocarbon thin film growth from methyl radicals and hydrogen atoms; (2) hydrocarbon thin film etching by ions and reactive neutrals; (3) plasma inactivation of bacteria; (4) plasma treatment of polymers; and (5) oxidation mechanisms during reactive magnetron sputtering of metal targets. All these mechanisms are unraveled by using a particle beam experiment to mimic the plasma–surface interface with the advantage of being able to control the species fluxes independently. It clearly shows that the mechanisms in action that had been described by Coburn and Winters [J. Appl. Phys. **50**, 3189 (1979)] are ubiquitous.

## I. INTRODUCTION

The interaction of low temperature plasmas with solid surfaces is at the core of many key technologies of the 21st century. Plasma treatment of thermolabile surfaces improves plastics to achieve wear protection or to improve barrier properties. Plasma coatings on metal substrates provide corrosion resistance against aggressive chemicals or serve as hard coatings to extend the lifetime of tools. Plasma etching of semiconductors is the workhorse in microelectronics and the driver for being able to follow Moore's law over the past few decades—the whole field of nanotechnologies relies heavily on the capabilities of plasma processes. All this is linked to the nonequilibrium character of plasmas, where the energy is invested in ionization and dissociation of species and not necessarily in the heating of the whole reaction volume. Thereby, the surfaces can remain cold while the surface reaction is triggered by incident reactive species. These heterogeneous surface reactions, however, are very complex because they may be governed by various synergisms and antisynergism among the particles, ions, electrons, photons, and radicals interacting at a growing or etched surface. The unraveling of these mechanisms directly in plasma experiments is difficult because all processes may occur simultaneously and any separation may remain ambiguous. This can be resolved by means of particle beam experiments using quantified beams of different species in an ultra-high-vacuum (UHV) environment with independent control of their fluxes and energies. Thanks to the independent control of particle sources, synergistic effects can be identified.

The most famous example of such a particle beam approach was presented by the pioneering work by Coburn and Winters on reactive etching of silicon.^1,2^ They showed that the etch rate of silicon or silicon oxide is significantly enhanced if reactive fluorine species and ions impact on the surface simultaneously. They coined the expression chemical sputtering to describe a process either where the chemical reaction creates an intermediate at the surface, which is then sputtered by the ions due to the lower surface binding energy, or where the ions damage the surface and make it accessible to the incident reactive neutrals. The most direct proof of the chemical sputtering process is the observation of a significant time delay, in the range of milliseconds, between an incident ion and the desorbing species caused by the out-diffusion of the etch products. Such a time delay was directly observed in modulated beam experiments for the etching of silicon by low-energy ions and fluorine atoms.^2^ The threshold for chemical sputtering is lower than that for physical sputtering since no momentum reversal of the incoming projectile has to take place. The absolute erosion yield is much higher because the erosion products do not only originate from the physical surface as in the case of physical sputtering but are also formed within the whole penetration range of the incident ions.

The ion-radical synergisms in silicon-containing plasmas are well studied. Here, we summarize similar experiments for an organic system, the growth and etching of hydrogenated amorphous carbon films, as well as for an inorganic system, the oxidation of metals.

(1) Hydrogenated amorphous carbon films are used with very different properties such as diamond like coatings as wear protection or they form naturally at the first wall of the nuclear fusion experiment when the graphite tiles interact with the hydrogen fusion plasma. The interaction of plasmas with hydrogenated amorphous carbon films is also an example for the atomistic processes during plasma treatment of polymers or for the interaction of plasmas with biological systems, which are organic interfaces on the atomistic scale. Finally, beam experiments have shed light on the effects of ions, metastables, and UV photons during plasma sterilization and chemical sputtering on spores.^3^
(2) Oxidation of a metal target during magnetron sputtering (MS) may lead to target poisoning and a strong hysteresis of the operating parameters voltage and reactive gas flow during MS. Therefore, the understanding of the effects of ions, radicals, reactive neutrals, and UV photons at a metal target surface is crucial for the development of reactive sputtering models.^4^ A prominent example is the ion-enhanced oxidation and the ion-induced secondary electron emission (SEE) from metal and metal oxide targets that can be described by an extension of Berg's magnetron sputter hysteresis model.^5,6^

All these systems and mechanisms are elucidated by using a particle beam experiment to mimic the plasma–surface interface. Thereby, the elementary input parameter of plasma-surface models can be uniquely measured. It clearly shows that the ion-neutral synergisms at the plasma–surface interface are ubiquitous, as already pioneered by Coburn and Winters.

## II. BEAM EXPERIMENT SETUP

**Fig. 1.**
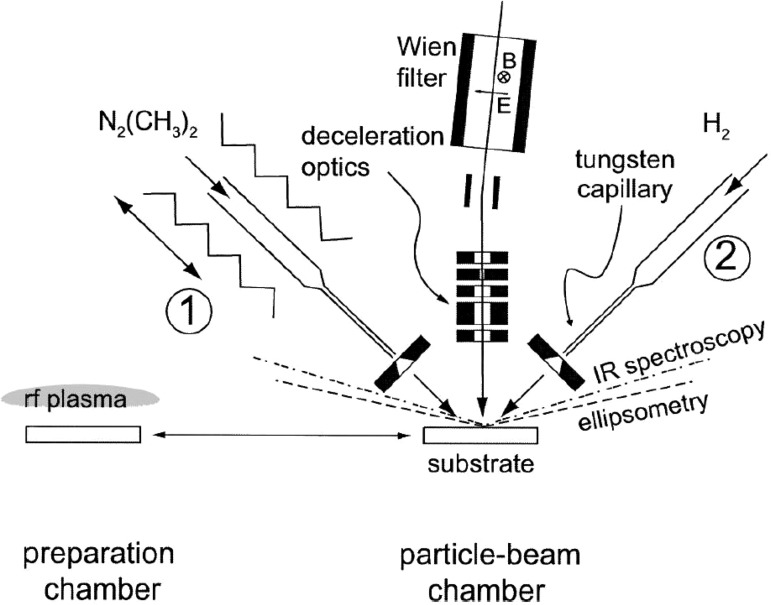
Sketch of an experimental setup used to perform particle beam experiments. The preparation chamber and the beam chamber are separated by a gate valve. The main components in the beam chamber are the ion gun system (ion source, Wien filter and deceleration optics), two radical beam sources, and two lines of sight for *in situ* diagnostics ellipsometry and FTIR. Reprinted with permission from Jacob *et al*., Rev. Sci. Instrum. **74**, 5123 (2003). Copyright 2003 AIP Publishing (Ref. 7).

Generally, beam experiment setups are separated into a particle beam chamber and a load-lock for the samples. The samples are then transferred into the particle beam chamber without breaking vacuum. The particle beam reactor is an UHV chamber equipped with several particle sources for the production and irradiation of known fluxes of different species.^7,8^ Figure 1 shows a sketch of a beam experiment used by Jacob *et al*.^7^ Hot capillaries and Evenson (plasma) sources are used to generate atom and radical beams from molecular precursors. Ion guns may consist of ECR-based plasma sources with ion optics to obtain energetic ion beams or, more sophisticated, may consist of an ion source setup equipped with a Wien filter for mass selection, a beam decelerator, and a deflection system to discriminate fast neutrals from charge exchange collisions. The radical and ion beams interact with the sample usually at normal incidence or with an angle of incidence of 45°. A base pressure in the 10^−7^ Pa range is reached after bake out. The working pressure is usually comprised within 10^−3^ and 3 × 10^−2 ^Pa, depending on the gas throughput.

A large variety of *in situ* diagnostics are used in beam experiments to monitor the heterogeneous surface processes during particle bombardment. The optical properties of the samples during beam irradiation can be determined by optical *in situ* real time ellipsometry. On the other hand, Fourier-transform infrared spectroscopy (FTIR) in the reflection mode provides the density of active chemical groups. Hence, creation or etching rates of these chemical groups shed light on the chemical state of the sample surface. Mass variation rates in real time are obtained using a quartz crystal microbalance (QCM) so that surface coating and sputtering can be modeled through flux balance equations including sputter yields and sticking coefficients. The yields of SEE can be measured using a special electrostatic collector consisting of biased coaxial electrodes. Besides, classical *in situ* diagnostics to provide the chemical composition and crystalline structure data, like x-ray photoelectron spectroscopy (XPS), Auger electron spectroscopy and reflected high energy electron diffraction are also usually employed during beam experiments. The composition of the particle beams can be characterized by quadrupole mass spectrometry. The ion flux and energy distribution of the ion beams are measured routinely using a Faraday cup and a retarding field energy analyzer, respectively.

## III. EXAMPLES AND DISCUSSION

### A. Hydrocarbon thin film growth from CH_3_ and H

**Fig. 2.**
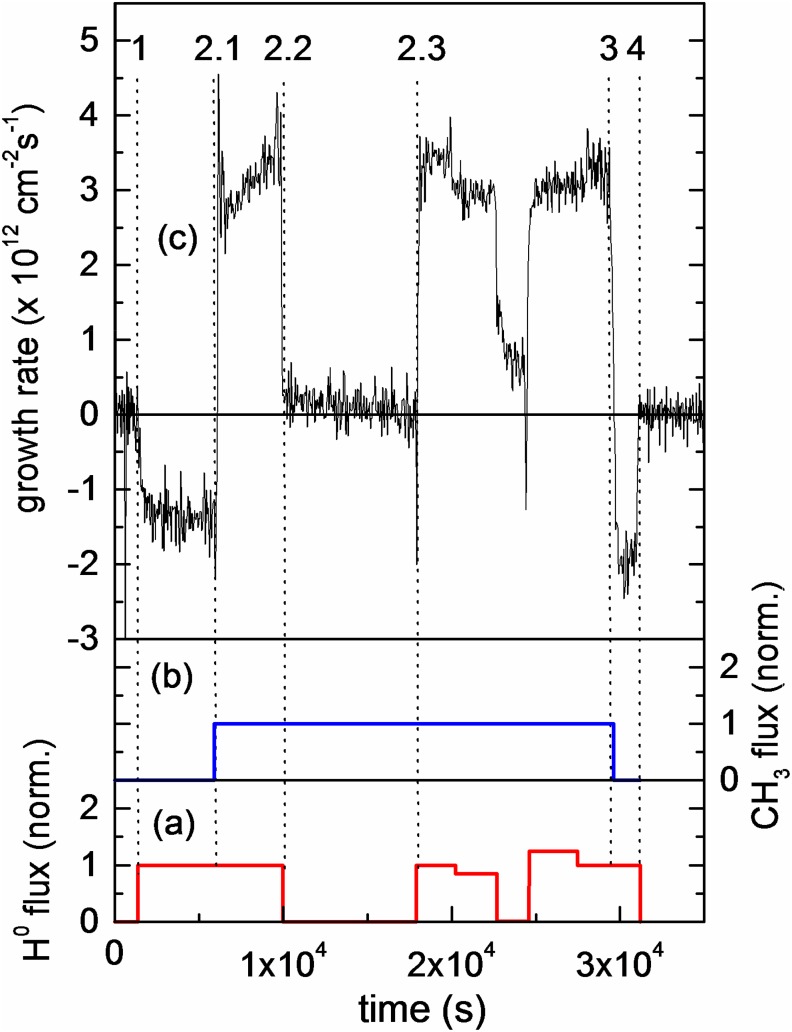
(Color online) (a) Variation in the impinging flux of H radicals normalized with 10^16 ^cm^−2^ s^−1^. (b) Variation in the impinging flux of CH_3_ radicals normalized with 2.5 × 10^15 ^cm^−2^ s^−1^. (c) Variation in the growth and/or etch rate during exposure of the a–C:H film to the CH_3_ and H beams. The numbers indicate changes in the beam parameters during the experiment. Reprinted with permission from von Keudell *et al*., Appl. Phys. Lett. **76**, 676 (2000). Copyright 2000 AIP Publishing (Ref. 16).

Amorphous hydrogenated carbon (a-C:H) films are frequently used for wear-resistant applications. They are deposited from glow discharges using a hydrocarbon precursor gas such as methane or acetylene. In the case of methane as the source gas, atomic hydrogen and CH_3_ radicals represent the dominant growth species.^9,10^ Diamond deposition is a variant of carbon deposition from discharges using a mixture of a few percent methane in hydrogen. It is believed that the microscopic growth mechanism consists of the adsorption of CH_3_ at free surface sites, which are created via the abstraction of surface-bonded hydrogen by incoming atomic hydrogen.^11–15^ Such a plasma process is investigated in a beam experiment using quantified beams of H atoms and CH_3_ radicals, as illustrated by the growth or etch rate in Fig. 2(c) for the different combinations of H and the CH_3_ flux shown in Figs. 2(a) and 2(b). One can clearly see that the growth rate is only high when both the species interact with the surface simultaneously. The sticking coefficient of CH_3_ under the “hydrogen beam on” condition is 10^−2^. At point 2.2, the atomic hydrogen beam is switched off and the growth rate drops significantly although the CH_3_ radical flux remains constant. The sticking coefficient under “H beam off” conditions is 10^−4^. This proves that CH_3_ adsorption is strongly enhanced by a simultaneous flux of atomic hydrogen.

The synergism between CH_3_ and H atoms explains very well the growth rate of amorphous hydrogenated carbon thin films and that of diamond deposition. It was also shown, however, that even without any activation of the surface, the sticking coefficient of CH_3_ is 10^−4^. This result had dramatic consequences for the design of future nuclear fusion reactors. For a long time, the first wall of a nuclear fusion experiment consisted of graphite tiles due to their heat resistance and compatibility with a hydrogen fusion plasma at millions of K. If a carbon atom enters such a fusion plasma due to sputtering of the first graphite wall, the core fusion plasma performance is not deteriorated. This is in contrast to the use of metals with high nuclear charge as the first wall. In a future nuclear fusion reactor, the hydrogen isotopes tritium and deuterium will be used. Due to safety reasons, an upper overall limit for the tritium content in the nuclear machine has to be ensured. Tritium retention may occur either in the form of the gas or bonded in surface layers or dissolved in the metal surfaces. The interaction of a tritium-containing fusion plasma will eventually cause the formation of CT_3_ when interacting with the graphite tiles. These CT_3_ radicals are expected to also have a low sticking coefficient of 10^−4^ and may survive many wall collisions and are, therefore, able to reach very remote locations of a nuclear fusion reactor. This corresponds to a permanent retention of tritium because deposited C:T layers cannot easily be removed in remote flanges or pump ducts. Consequently, the allowance limit for the tritium content in a nuclear fusion reactor may be reached because eventually all tritium is bound in C:T layers at inaccessible locations. This is not tolerable so that eventually the nuclear fusion community abandoned the predominant use of carbon as the first wall material and switched to tungsten and beryllium for the next generation of fusion reactors.

### B. Hydrocarbon thin film etching by ions and reactive neutrals—Chemical sputtering

**Fig. 3.**
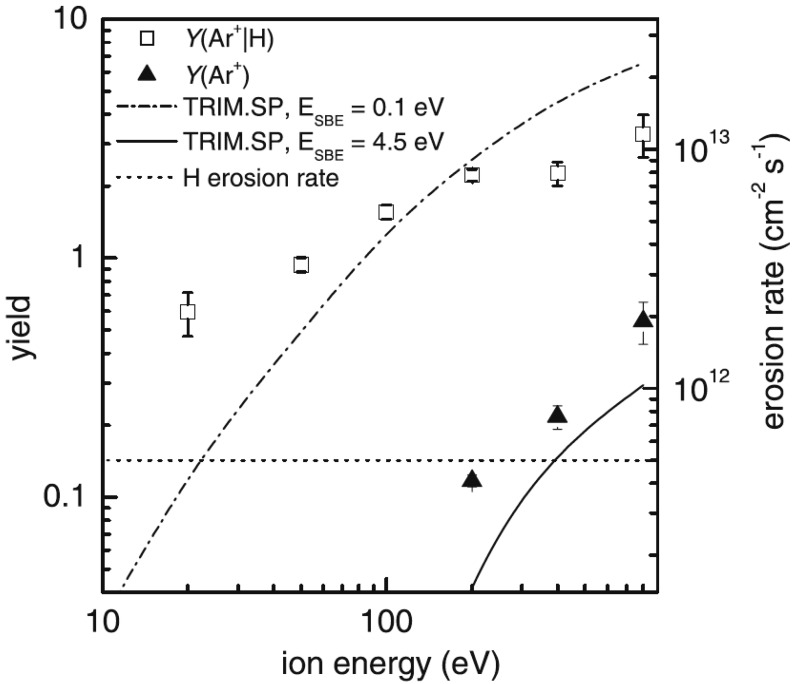
Erosion yield *Y*(Ar^+^) for physical sputtering of the a-C:H film by Ar^+^ ions (solid symbols) and *Y*(Ar^+^|H) for chemical sputtering by a simultaneous flux of Ar^+^ ions and H atoms (open symbols). The atom-to-ion flux ratio is 400, and the substrate temperature is 320 K. The dashed-dotted and solid lines are carbon erosion yields from TRIM.SP calculations. The dotted line refers to the absolute erosion rate by H atoms only (Ref. 18).

Synergisms at the plasma-surface interface do play a role not only in film growth of a-C:H but also in etching. It is known that a-C:H film erosion by hydrogen ions can occur even at low substrate temperatures and low ion energies. This is surprising because chemical erosion by H atoms can be excluded since it is a temperature activated process, which is negligible at the low temperatures. Also, physical sputtering can be excluded because the ion energies are below the threshold for this sputtering mechanism.^17^ This can be resolved by using beam experiments exposing a-C:H layers to argon ions and a beam of hydrogen atoms. Figure 3 illustrates that sputtering by argon ions is drastically enhanced if an additional flux of atomic hydrogen is present. The erosion rate is by far higher than the sum of the etching rates for atomic hydrogen and Ar^+^ ions only. The prediction for chemical etching is shown as the dotted line in Fig. 3, and the prediction for physical sputtering is shown as the solid line in Fig. 3. In addition, significant film etching is observed even below the threshold energy for sputtering by argon ions only. A direct analysis of the surfaces reveals that the hydrogen content at the surface remains high in the case of a simultaneous flux of Ar^+^ ions and H atoms, while it decreases in the case of a bombardment with Ar^+^ ions only. This indicates that the additional flux of atomic hydrogen leads to the incorporation of hydrogen in the film, thereby compensating the release of bonded hydrogen caused by the incident Ar^+^ ions. The large difference between Y (Ar^+^) and Y (Ar^+^|H) further shows that the C:H film surface with a high hydrogen content is more susceptible to sputtering.

In the case of physical sputtering, the momentum of an incident projectile has to be reversed before it transfers kinetic energy to a surface atom or group to overcome the surface binding energy. In contrast, film sputtering at ion energies below the threshold for physical sputtering is explained by the process of “chemical sputtering,” as defined in detail by Winters *et al*.^2,19^ According to this definition, chemical sputtering of C:H films by Ar^+^ ions and H atoms can only occur if ions and H atoms interact simultaneously with the surface: incident ions create broken bonds within the collision cascade. These broken bonds are instantaneously passivated by the abundant flux of atomic hydrogen. This leads to the formation of stable hydrocarbon molecules underneath the surface. They finally diffuse to the surface and desorb.

Ion-induced surface processes not only are relevant for chemical sputtering but may also trigger film growth:^20^ incident ions displace surface bonded species within the collision cascades and create thereby dangling bonds at which incident radicals may chemisorb. Such a process is especially important for film growth at low ion energies, where the physical sputtering rate can be negligible and the incident radicals have a low sticking coefficient. Film growth is, therefore, very susceptible to any additional surface activation. A good agreement between measurements and modeling was found by Hopf *et al*. for a-C:H film growth,^21^ who showed that a helium ion beam activated the surface and facilitated the chemisorption of incident CH_3_ radicals at dangling bonds with a probability of unity. The best agreement was found if one also includes the recombination of adjacent dangling bonds at the surface corresponding to the transition between sp^3^ and sp^2^ bonds. The quantitative interpretation of ion-induced film growth may be, however, more difficult because incident ions not only cause chemical sputtering and surface activation but also modify the plasma exposed surface within the penetration depth of the ions. For example, an intense ion bombardment not only depletes the surface of hydrocarbon films from hydrogen, which makes them more resistant against chemical and physical sputtering, but also reduces the ion-induced formation of dangling bonds as adsorption sites for incident radicals. Since both the rates of ion-induced etching and ion-induced film growth decrease, it remains difficult to isolate one effect from the other.

### C. Hydrocarbon thin film etching—Chemical sputtering of spores

**Fig. 4.**
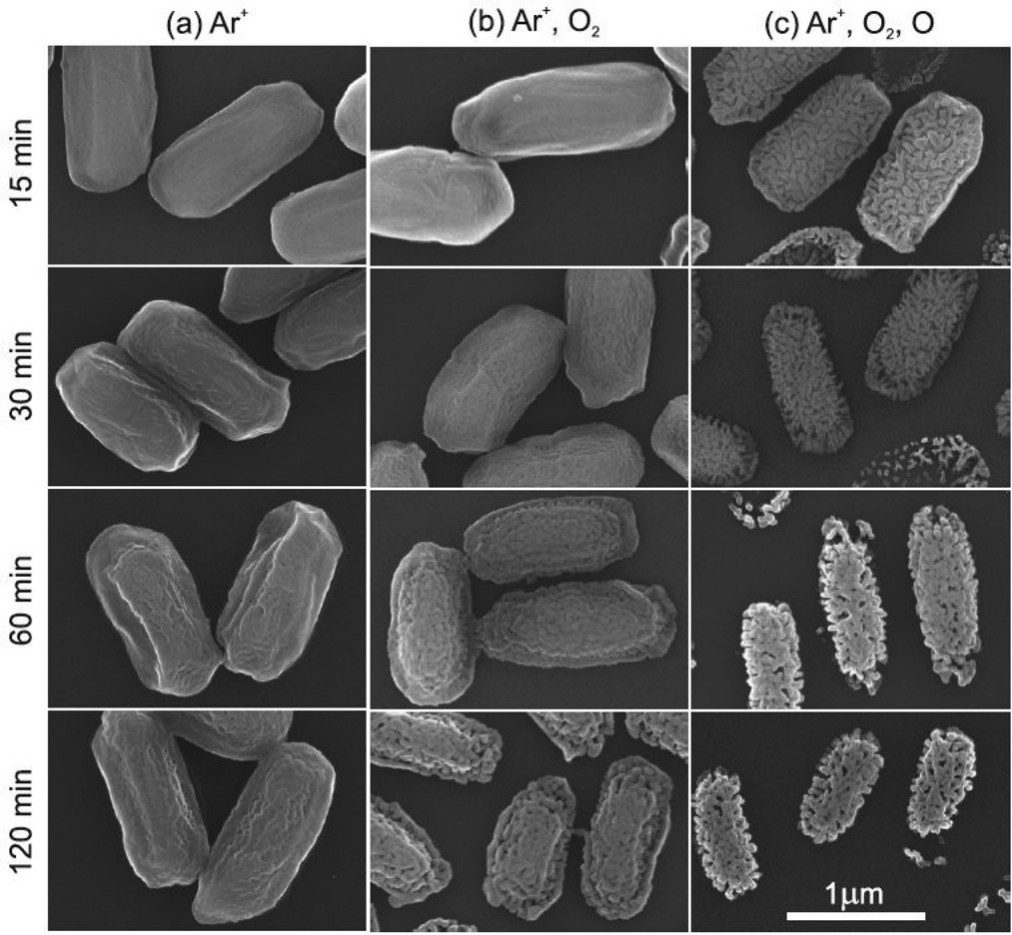
SEM micrographs of *B. atrophaeus* exposed between 15 and 120 min to (a, first column) argon ions ($j_{{\text{Ar}}^{+}}$ = 1.8 × 10^14 ^cm^−2^ s^−1^) at 200 eV, to (b, second column) a combined flux of argon ions ($j_{{\text{Ar}}^{+}}$ = 1.8 × 10^14 ^cm^−2^ s^−1^) at 200 eV and O_2_ molecules ($j_{O_{2}}$ = 4.5 × 10^15 ^cm^−2^ s^−1^), and to (c, third column) a combined flux of argon ions ($j_{{\text{Ar}}^{+}}$ = 1.6 × 10^14 ^cm^−2^ s^−1^) at 200 eV, O_2_ molecules ($j_{O_{2}}$ = 1.5 × 10^17 ^cm^−2^ s^−1^) and O atoms (*j*_O_ = 2.4× 10^15 ^cm^−2^ s^−1^). Reprinted with permission from Raballand *et al*., J. Phys. D: Appl. Phys. **41**, 115207 (2008). Copyright 2008 IOP (Ref. 3).

The ion-neutral synergism during the etching of a-C:H layers by incident argon ions and hydrogen atoms can also serve as a model system to understand the interaction of plasmas with biological systems in plasma sterilization. Plasma sterilization is a modern technology to inactivate especially very resistant germs^22–24^ using very often argon as plasma gas with admixtures of nitrogen, hydrogen, or oxygen.^25–27^ The main advantage of plasma sterilization is its ability to sterilize also thermolabile medical tools made of plastics. The validation of a sterilization technique is usually based on the proof to inactivate endospores of bacteria, which are known to be a very resistant biological system. The inactivation itself is caused by UV photons that induce DNA strand breaks within seconds of exposure to a plasma.^28,29^ The sterilization efficiency is, however, in realistic scenarios largely reduced because the UV radiation is shadowed by multilayered stacks of spores, which makes an additional plasma-induced chemical or physical etching of the biological systems essential. Such a plasma etching, however, needs to be mild enough not to harm the object being sterilized. The effect of oxygen atoms, oxygen molecules, and incident argon ions on the ability to inactivate and to etch *Bacillus atrophaeus* spores is determined in the beam experiment. Figure 4(a) indicates by SEM micrographs that endospores exposed to Ar^+^ ions at 200 eV show only a slightly altered surface texture [see Fig. 4(a)]. Apparently, Ar^+^ ion bombardment at 200 eV does not cause any significant erosion of the spore coat. This can be explained by the energy dependence of the physical sputtering yield *Y* (Ar^+^) of hydrocarbon compounds by argon ions. The physical sputtering process becomes only significant for ion energies well above 200 eV.^30^ Endospores exposed to a simultaneous flux of argon ions and oxygen molecules exhibit, however, a very different appearance [see Fig. 4(b)]. Although the absolute size of the spores did not change noticeably, the spore coat became porous, showing etch channels with an estimated depth of approximately 100 nm after 60 min. The sputtering yield can be estimated from the known argon ion flux of 1.8 × 10^14 ^cm^−2 ^s^−1^ to be *Y* (Ar^+^|O_2_) ≈ 1, which is well above the expected value for a pure physical sputtering process [*Y* (Ar^+^) ≈ 0.06 for an Ar^+^ ion energy of 200 eV].

The high sputtering yield is again explained by the simultaneous impact of ions and oxygen molecules leading to the process of chemical sputtering.^20^ The repeated ion-induced bond breaking followed by their reaction with oxygen molecules leads, below the surface, to the formation of presumably CO, CO_2_, and H_2_O as volatile components. These reaction products diffuse to the surface and desorb.^1^ As a result, etching occurs, making plasma sterilization of multilayered biological samples a viable method for the health care industry.

### D. Plasma treatment of polymers—Chemical sputtering

The interaction of ions and radicals with a-C:H films is also a model system for plasma-surface treatment of polymers for either lithography purposes (photoresist) or hydrocarbon removal (plasma cleaning).^31–34^ For example, argon plasmas are extensively used to activate polymer surfaces in order to enhance their adhesion with subsequently deposited layers.^31^ On the other hand, the addition of oxygen to the gas mixture facilitates the generation of polar groups on the treated surface, thereby increasing the surface energy.^35–37^ Another expected effect of oxygen in the plasma is an increase in the etching rate due to chemical sputtering of the hydrocarbon network, which produces bond scission reactions leading to the creation and desorption of volatile CO_2_ and H_2_O molecules.

**Fig. 5.**
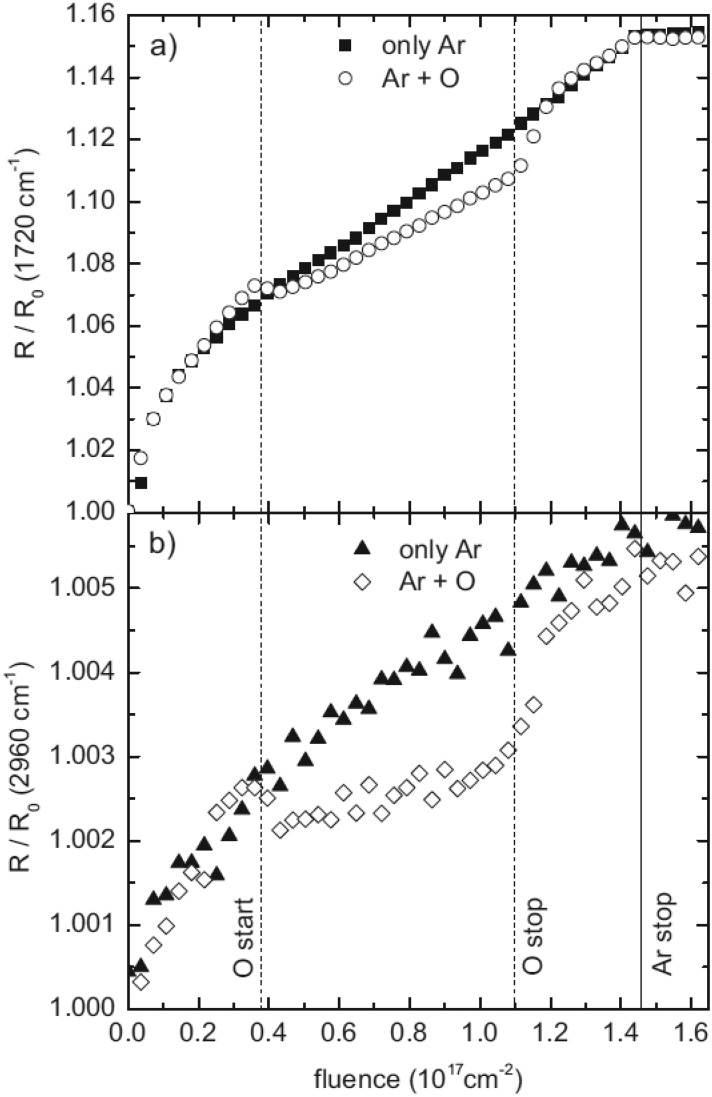
FTIR normalized spectra *R*/*R*_0_ for the removal of C=O groups (a) and CH_x_ groups (b) in an experiment exposing PET to argon ions only (solid symbols) and argon ions and oxygen atoms (open symbols). The oxygen beam is added after an accumulated ion fluence of 0.4 × 10^17 ^cm^−2^ (marked as the dashed line, “O start”) and stopped at an accumulated ion fluence of 1.1 × 10^17 ^cm^−2^ (marked as the dashed line, “O stop”). The argon beam is stopped at an accumulated ion fluence of 1.45 × 10^17 ^cm^−2^ (marked as the solid line, “Ar stop”). The argon ion energy is 200 eV. Reprinted with permission from Groβe-Kreul *et al*., Plasma Processes Polym. **10**, 225 (2013). Copyright 2013 Wiley (Ref. 38).

Surface modifications of polyethylene terephthalate (PET) and polypropylene (PP) by Ar ions, oxygen atoms and molecules, and UV photons have been investigated in beam experiments with known fluxes of argon ions and oxygen neutrals to mimic plasma treatment: (1) in the case of PET, the addition of oxygen to the incident argon ion flux does not enhance the etching rate but only changes the surface composition as evidenced by *in situ* FTIR analysis and subsequent contact angle measurements.^38^ Figure 5 shows how the normalized infrared reflectivity R/R_0_ for the removal of the C=O groups (1720 cm^−1^) and CH_x_ groups (2960 cm^−1^) is affected by the different bombarding conditions. In fact, the sputtering of PET is dominated by chemical sputtering with an internal source of reactive species. As a consequence, any addition of an external source of reactive species cannot alter the already high sputter yield, and the addition of the oxygen beam does not affect the etching rate. The yields at low and high ion energies have been compared with transport of ions in matter (TRIM) calculations. The measured yields remain higher than the TRIM yields irrespective of the ion energy because of the presence of the intense internal source of reactive species. This source of reactive species is represented by the displaced hydrogen and oxygen atoms in a collision cascade.^38^

(2) Plasma treatment of PP shows a different scenario. The source of internal reactive species is reduced since no oxygen is present. On the other hand, the structure of PP is more sensitive to UV photons than in the case of PET, which leads to a synergistic effect between argon ions and UV radiation: the sputter yield by Ar^+^ ions is maximized at 200 eV.^4^ This combined action of ions and photons toward a very efficient etching is consistent with the measured IR spectra of the modified top layer, which fits perfectly with untreated PP and indicates that net etching without chemical conversion takes place at this ion energy.^39^

The chemical sputtering of PP films by combined bombardment of Ar ions, UV photons, and oxygen neutrals is discussed in the following. The cross-linking on PP produced by the UV photons, whose penetration depth is some tens of nanometers, is in competition with the etching induced by the same photons and the incident argon ions. Also, the dense top-layer caused by energetic ion bombardment (ca. 2–3 nm thick of amorphous carbon) introduces an additional barrier that reduces chemical sputtering by oxygen and argon ions at the PP surface. This explains why the etch rate only increases upon addition of oxygen at low ion energies (ca. 20 eV), whereas the rate is found to be approximately constant at higher ion energies (over 200 eV). This explanation based on a hardening effect at high ion energies is in agreement with *in situ* FTIR measurements, which show a cross-linking of the polymer due to selective etching of methylene groups and/or exomethylenic bond formation by oxygen atoms.^39^

### E. Oxidation mechanism during reactive magnetron sputtering

Finally, beam experiments can also be used to analyze inorganic systems, such as the interaction of plasmas with metal surfaces during magnetron sputtering of metals or oxides and nitrides. Plasma deposition processes using reactive magnetron sputtering of metals are of major importance for many present day technologies.^40,41^ The addition of oxygen to the discharge leads to the formation of a compound metal oxide on the growing film surface. Also, it causes hysteresis effects in sputtering processes due to the oxidation of the target surface, whose state can be described in terms of rate equations providing fundamental parameters like sticking coefficients and sputter yields (Berg model).^42,43^

**Fig. 6.**
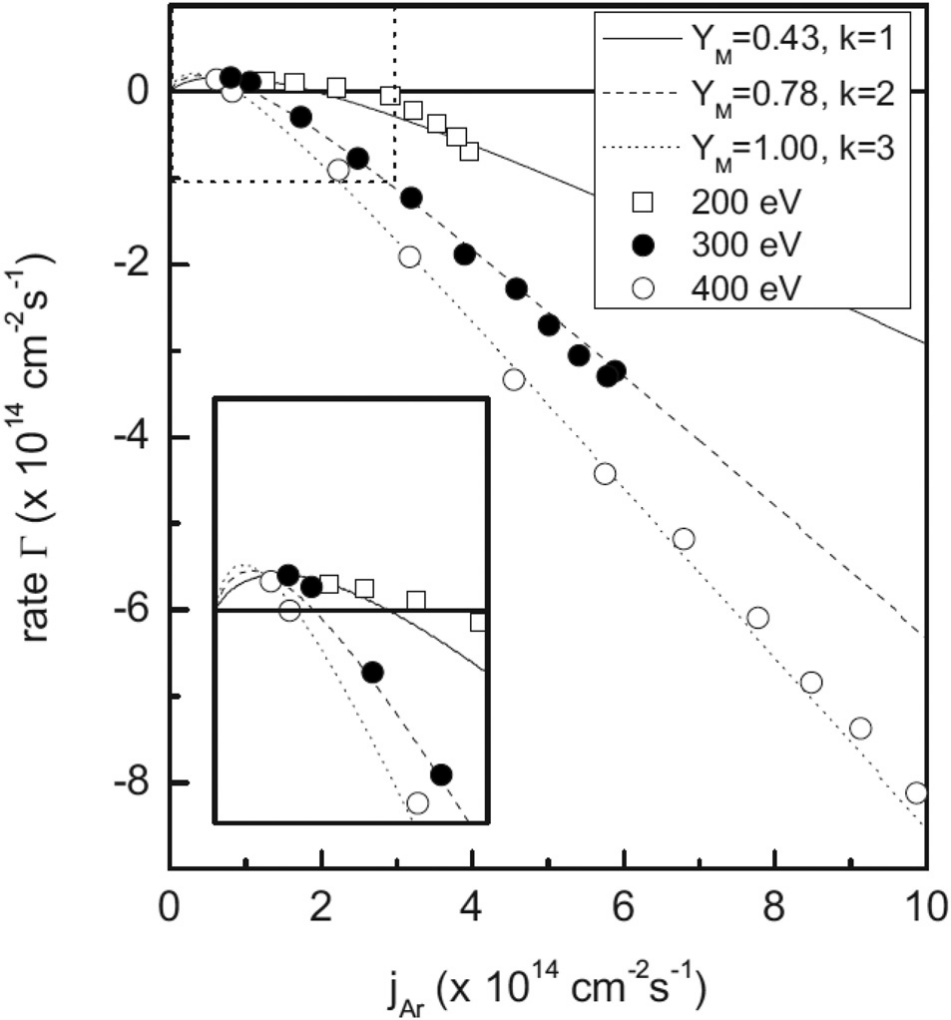
Dependence of the oxygen uptake/removal rate of an Al-covered QCM, as being exposed to an oxygen molecule flux of 4.1 × 10^15 ^cm^−2^ s^−1^ and varying argon ion flux and energy. The solid, dashed, and dotted lines refer to the fitted model. Here, an energy dependence for the knock-on implantation is assumed by varying the knock-on parameter, *k*. The inset shows the comparison of data and the model for small argon ion fluxes. Reprinted with permission from Kuschel and von Keudell, J. Appl. Phys. **107**, 103302 (2010). Copyright 2010 AIP Publishing (Ref. 5).

The fundamental surface processes in the Berg model are oxygen chemisorption, reactive ion implantation, sputtering of metal and the oxide, and knock-on implantation of oxygen by the ion bombardment to explain the ion-enhanced oxidation of aluminum during reactive magnetron sputtering.^5^ The sticking coefficient of ground-state oxygen atoms and molecules on aluminum is relatively low (0.015). However, the measured effective sticking values during simultaneous bombardment of Al with Ar^+^ beams and O/O_2_ species remain significantly larger than zero in steady state conditions. Such a phenomenon is a signature of ion-enhanced chemical oxidation by oxygen displacement, which can be linked to the implantation of oxygen due to the impact of energetic argon ions. The probability of this event is quantified by the so called knock-on implantation coefficient.^44^ Figure 6 shows the influence of the sputter rate on the argon flux at different ion energies, and the corresponding model fits with different knock-on coefficients. The synergistic effect of simultaneous irradiation with ions and oxygen is modeled with an extension of the Berg model, as illustrated by the lines in Fig. 6. This result proves the reliability of this model to study heterogeneous surface processes.

**Fig. 7.**
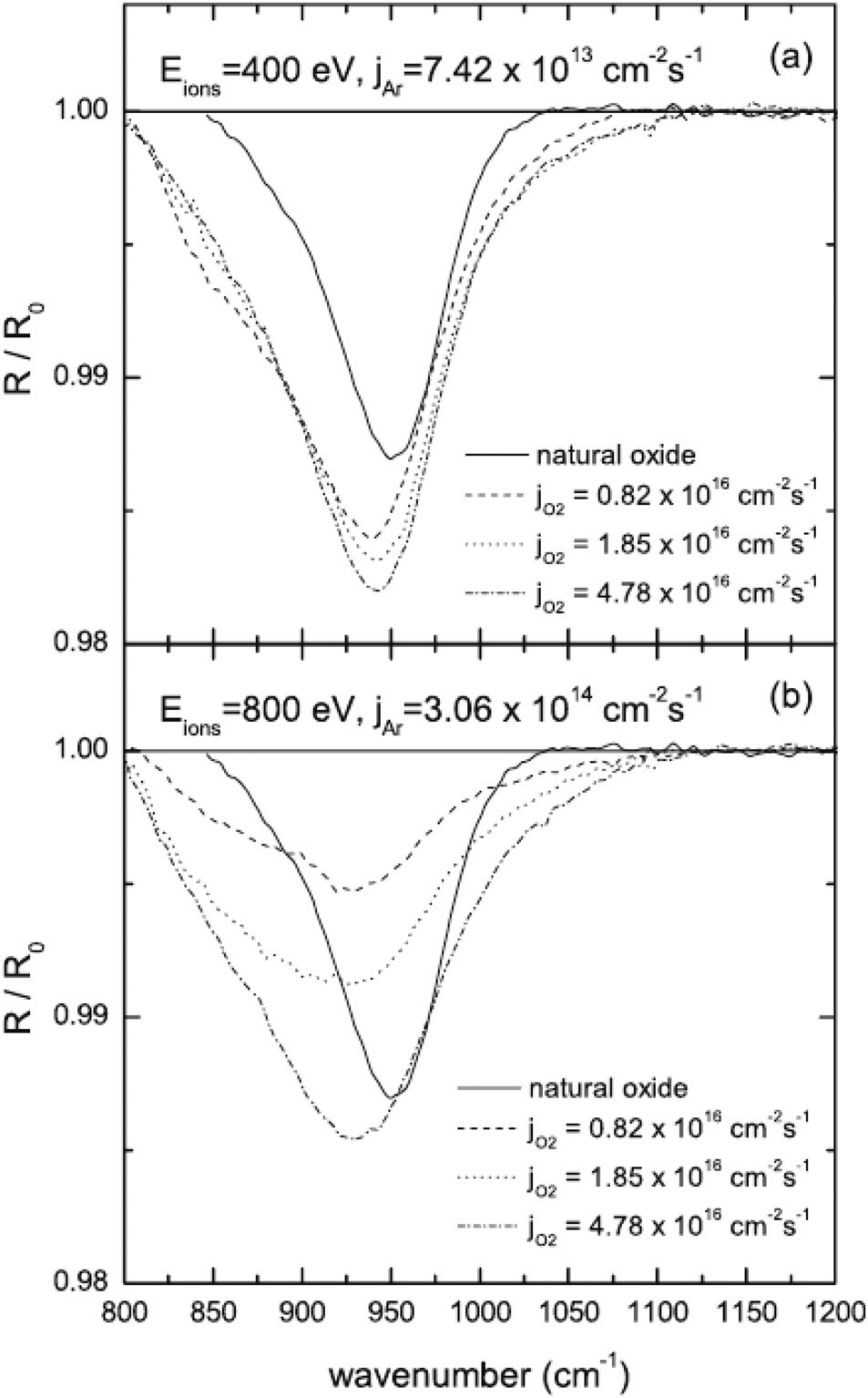
FTIR normalized spectra *R*/*R*_0_ during reactive sputtering of Al at (a) 400 eV and (b) 800 eV, at different oxygen fluxes. The solid line denotes the spectra if the oxygen source is used only at its maximum flow rate of 0.5 sccm. Reprinted with permission from Kreiter *et al*., J. Appl. Phys. **113**, 143303 (2013). Copyright 2013 AIP Publishing (Ref. 45).

The fact that the knock-on coefficient of oxygen turned out to be of the order of unity and higher reveals that surface activation by ion bombardment is a very efficient mechanism to increase aluminum reactivity with oxygen atoms and molecules. Parallel to QCM experiments, the knock-on implantation of oxygen onto the Al subsurface was investigated by *in situ* FTIR.^45^ There, the oxide absorption band from ion-bombarded oxidized Al surfaces was compared with the oxide absorption band of the Al surface thermally saturated with oxygen. The excess signal corresponding to the former case demonstrates the significant subplantation of oxygen atoms under the metal top layer during reactive sputtering of aluminum (Fig. 7). This result is consistent with the depth of the ion-enhanced oxidation of ion-treated Al surfaces measured using XPS and confirmed using the computer code transport of ions dynamics.^5^

In contrast, other metals like chromium show that knock-on implantation of oxygen atoms is a weaker oxidation mechanism compared to, for example, dissociative chemisorption, which is reflected by a much higher sticking probability of oxygen on chromium.^46^

The application of a retarding field on the samples by means of a counter-electrode provided the secondary electron yields of metals at low-medium Ar^+^ ion energies (500–2000 eV), which were of the order of ≈0.1.^6,47^ The addition of oxygen molecules to the beam reactor enhanced substantially the ion-induced emission of secondary electrons, with this effect being remarkable in the case of aluminum oxide. In general, this result was interpreted as the well-known effect of higher electron emission in oxidized surfaces. However, in addition, oxidized aluminum provided an energetic component of secondary electrons associated with Auger transitions, which abnormally increased the yield values over the unity.^48^

## IV. SUMMARY AND OUTLOOK

In this article, five examples of synergistic surface reactions at the plasma–surface interface are mimicked using beam experiments. Thereby, quantitative information on sticking coefficients, chemical sputtering yields, and secondary electron emission coefficients is derived. These input parameters for plasma modeling and plasma-surface modeling are of paramount importance because the heterogeneous surface reactions are usually the big “unknowns” in the description of technological plasma processes in general. Besides the determination of fundamental parameters, beam experiments are also able to elucidate mechanisms such as chemical sputtering, as it was introduced by the pioneering work by Winters. It is now known that such synergisms are ubiquitous in many systems and explain not only hydrocarbon film etching and growth but also the interaction of the plasma with bacteria and cells.

It will be the demand of the future to expand the beam experiment approach to many further systems to form a quantitative basis for the understanding and description of the plasma-surface interface. In the following years, the roadmap in beam experiments can be connected to further fields, other than the ones described in this article, interested in synergistic effects among diverse plasma species incident on solids:

(1) Sputtering phenomena on complex nonplanar surfaces, like on nanopatterns obtained by nanolithography techniques. Plasma micro-/nanopatterning could be thus monitored using beam experiments. Also, control of target defects, crystal phase, roughness, and porosity are required to mimic exactly the real surface processes during plasma exposure.
(2) Study of ion-surface interactions in two-dimensional materials like graphene for applications like functionalization and decoration. The role of the site occupied by, e.g., hydrogen on upper and lower surfaces and edges in, for example, nanoribbons, is the subject of intense research in plasma engineering that could be approached with beam experiments.^49,50^
(3) The existence of prebiotic molecules (e.g., glycine) exposed to ionized regions in the extraterrestrial environment defines the central topic in Astrobiology. The study of ion and electron beam irradiation of biomolecules will provide valuable information to explain the presence and the persistence of certain prebiotic elementary species in the outer space, which could be connected to the origin of life in the Universe.^51^
